# Circadian Clocks for All Meal-Times: Anticipation of 2 Daily Meals in Rats

**DOI:** 10.1371/journal.pone.0031772

**Published:** 2012-02-15

**Authors:** Ralph E. Mistlberger, Brianne A. Kent, Sofina Chan, Danica F. Patton, Alexander Weinberg, Maksim Parfyonov

**Affiliations:** Department of Psychology, Simon Fraser University, Burnaby, Canada; Pennsylvania State University, United States of America

## Abstract

Anticipation of a daily meal in rats has been conceptualized as a rest-activity rhythm driven by a food-entrained circadian oscillator separate from the pacemaker generating light-dark (LD) entrained rhythms. Rats can also anticipate two daily mealtimes, but whether this involves independently entrained oscillators, one ‘continuously consulted’ clock, cue-dependent non-circadian interval timing or a combination of processes, is unclear. Rats received two daily meals, beginning 3-h (meal 1) and 13-h (meal 2) after lights-on (LD 14∶10). Anticipatory wheel running began 68±8 min prior to meal 1 and 101±9 min prior to meal 2 but neither the duration nor the variability of anticipation bout lengths exhibited the scalar property, a hallmark of interval timing. Meal omission tests in LD and constant dark (DD) did not alter the timing of either bout of anticipation, and anticipation of meal 2 was not altered by a 3-h advance of meal 1. Food anticipatory running in this 2-meal protocol thus does not exhibit properties of interval timing despite the availability of external time cues in LD. Across all days, the two bouts of anticipation were uncorrelated, a result more consistent with two independently entrained oscillators than a single consulted clock. Similar results were obtained for meals scheduled 3-h and 10-h after lights-on, and for a food-bin measure of anticipation. Most rats that showed weak or no anticipation to one or both meals exhibited elevated activity at mealtime during 1 or 2 day food deprivation tests in DD, suggesting covert operation of circadian timing in the absence of anticipatory behavior. A control experiment confirmed that daytime feeding did not shift LD-entrained rhythms, ruling out displaced nocturnal activity as an explanation for daytime activity. The results favor a multiple oscillator basis for 2-meal anticipatory rhythms and provide no evidence for involvement of cue-dependent interval timing.

## Introduction

Nocturnal rats with free access to food typically eat numerous small meals throughout the night, but can readily adapt to restricted feeding schedules in which food is provided for only a few hours each day in the middle of the light period. Adaptation involves shifting of circadian cycles of clock gene expression and clock-controlled rhythms in most peripheral organs and the gradual emergence of a robust bout of locomotor activity anticipating mealtime by 1–3 h [1], [2]. Food anticipatory activity induced by such feeding schedules has been conceptualized as a circadian rhythm driven by a food-entrained circadian oscillator (FEO) separate from the master light-entrainable circadian pacemaker located in the suprachiasmatic nucleus (SCN). This entrained oscillator model can account for properties of food anticipatory rhythms, including persistence for several cycles during total food deprivation, gradual resetting following meal shifts, changes in the onset of anticipation when the period of the feeding schedule is shortened or lengthened within the 22–29 h range, failure of anticipation to emerge or remain synchronized if feeding schedules fall outside of this ‘circadian’ range, and persistence following SCN-ablation [1], [3]–[5]. The model is parsimonious to the extent that food anticipatory behavior, like daily rhythms entrained to LD cycles, can be explained as a simple oscillator-driven rest-activity cycle without recourse to higher order cognitive processes.

A dual-FEO model has been suggested to account for the ability of rats and mice to anticipate two daily meals separated by 5 h or more, and the apparent failure of rats to anticipate three daily meals [6]–[8]. In these studies, some rats with SCN-ablations could at least temporarily anticipate two daily meals with different period lengths (e.g., concurrent 23.75-h and 24.0-h feeding schedules) [6], [8]. Anticipation tended to be unstable, and during total food deprivation tests only a single bout of activity spanning the two mealtimes was evident, possibly because the mealtimes were too close during the test. This caveat aside, the results provide at least tentative support for the idea that circadian oscillators driving food anticipation can be configured as a single FEO or as two independently entrainable FEOs. Precedence for the concept of a 2-oscillator structure is provided by the light-entrainable SCN pacemaker, for which there is behavioral, electrophysiological and molecular evidence [9]–[11].

Anticipation of two daily meals could also be accounted for by the concept of a single FEO functioning as a so-called ‘continuously consulted clock’. A consulted clock provides a continuous readout of circadian phase, which can be used to recognize and record the time of occurrence of daily events, such as meals. To be useful, the clock must be synchronized to local time, so that its phase predicts daily events. The use of circadian oscillators as consulted clocks is believed to underlie sun compass orientation in various insects and birds that forage or migrate over distance [12]. There is also evidence that birds [13], [14], rats [15]–[17] and mice [18] can use circadian phase as a discriminative cue for time-place learning (e.g., associating a time of day with a unique feeding location or operant response). There is some evidence that rats can accomplish this without a SCN [16], raising the possibility that anticipation of two daily meals could be mediated by a single FEO serving as a consulted clock, enabling animals to anticipate one or more meals at any arbitrary phase. This type of mechanism would require additional cognitive processes for linking representations of clock phase to event memories, and for generating rules to enable anticipation of two daily meals with different periodicities in the circadian range [6], [8].

A circadian clock basis for anticipation of daily meals has been challenged by the persistence of food anticipatory rhythms in mice with mutations of known circadian clock genes [19], [20]. This may indicate the existence of novel molecular or network processes for generating circadian behavioral rhythms, or the availability of non-circadian mechanisms such as interval timers that can compensate when circadian mechanisms are disabled [21]–[22]. Interval timers have been conceptualized as neural stopwatches for measuring elapsed time between events, to explain the properties of anticipatory behaviors when food rewards are provided at fixed intervals in the seconds to minutes range, or when rewards are preceded by an external stimulus, as in Pavlovian conditioning paradigms [17], [21], [23]. These properties are distinct from those of rhythms generated by entrained oscillators; the anticipation ‘rhythm’ does not persist if the cue or reward are omitted, resets immediately rather than gradually when the cue or reward cycle are shifted, and exhibits proportionality between the average duration of anticipatory responding and the duration of the interval being timed [formally, the standard deviation of the response distribution is a constant proportion of the duration of the interval being timed, known as the ‘scalar’ property) [23]. Violations of stopwatch and scalar properties have been noted in short interval timing [17], [24], suggesting alternative models, but interval timers that measure elapsed time between events have generally been discounted as a possible mechanism for inducing anticipatory rhythms under circadian feeding schedules.

Nonetheless, a contribution of interval timing to single or 2-meal anticipatory rhythms has not been ruled out. It has recently been suggested that “*it is likely that animals learn the time of (daily) feeding with respect to a light-entrainable oscillator, food entrainable oscillator(s), interval timers triggered by light onset or offset and perhaps an interval timer that records the duration of one meal to the next*” [21]. There is little direct evidence by which to evaluate this proposition as it pertains to rodents, as most studies of circadian food anticipation have reported on the presence of anticipation, without quantifying anticipation parameters such as duration, magnitude and variability, in the presence and absence of events, such as lighting transitions and meals, that could actuate interval timers and influence anticipatory behavior. The first study to explicitly examine a role for interval timing in circadian food anticipation found that the presence of an auditory tone beginning 2-h or 4-h prior to a daily meal resulted in a pause in anticipatory responding (lever pressing), thereby delaying the rise time and reducing the terminal peak of the anticipation waveform [25]. A more recent study observed that rats initiated anticipatory operant behavior earlier prior to a single daily meal delivered 7-h after lights-off compared to a meal delivered 3-h after lights-off, and that the duration of anticipation was a constant proportion of the interval between lights-off and mealtime [26]. This evidence for the scalar property suggests that rats may measure elapsed time between a LD transition and a predictable mealtime 3–7 h later. However, the study did not include a food deprivation test in constant dark (DD) to confirm that the differences in anticipation duration were dependent on external time cues. If the differences were to persist in the absence of LD and feeding cues, then these could not be related to psychophysical properties of interval timing, and would have to involve other factors, such as the different phases of the SCN pacemaker at which the meals occur.

A quantitative model of daily meal timing in rats will need to incorporate contributions made by circadian, interval timing, homeostatic, and other processes, where these can be empirically substantiated. We report here the results of experiments designed to evaluate the role of interval timing and circadian oscillators in anticipation of two daily meals in rats. Sufficient work has been done to establish that rats can anticipate two daily meals in the light or dark period, and that anticipation can persist in DD and without a SCN [6], [7], [15], [16], [27]–[31]. However, analyses in these studies where qualitative rather than quantitative, and thus provide a limited basis for evaluating potential contributions of interval timing mechanisms, or for discriminating between single and multiple circadian oscillator models. In the present study, food was provided for 1-h twice each day during the light period, at dissimilar intervals relative to lights-on and to prior mealtime. The onsets of the two daily bouts of anticipatory locomotor activity were quantified in LD, on meal omission days conducted in LD and DD, and following an acute shift of one mealtime. The results provide no evidence for a contribution of interval timing under the conditions of this study, but do provide support consistent with a dual-FEO model.

## General Methods

### Animals and Apparatus

Adult male Sprague Dawley rats (450–550 g; Charles River, PQ) were housed individually in clear plastic cages (40.6 cm×50.8 cm×21 cm) equipped with a 35.5 cm running wheel and a metal cage top holding a water bottle and food hopper (Lafayette Instruments, IN, USA). Each cage was housed in an isolation box (Lafayette Instruments) with controlled lighting (LD 14∶10, white LEDs, ∼30 Lux) and an exhaust fan. A long (14 h) photoperiod was used so that 2 daily meals could be scheduled during the light period, when anticipation is easy to detect in wheel running activity, with an intermeal interval sufficient to unambiguously distinguish separate food anticipation bouts during meal omission and meal shift tests. After the first experiment, the cages were moved to open cabinets, 2 cages per shelf, and a food bin was attached to the outside of each cage, accessible via a 4 cm square window. Temperature in the vivarium was maintained at ∼22°C. Wheel revolutions were detected by magnetic switches and food-bin activity by infra-red beam breaks. Activity counts were summed and stored at 1-min intervals using the Clocklab data acquisition system (Actimetrics, IL USA). All animal work was conducted according to guidelines established by the Canadian Council on Animal Care and was approved by the University Animal Care Committee at Simon Fraser University (permit number 732P95).

### Data analysis

For visual inspection, activity data were collapsed into 10 min bins plotted in the standard ‘actogram’ format using Circadia (Dr. T.A. Houpt, Florida State University), and as 24-h waveforms averaging one or more days across animals within groups, using Prism 5.0 (Graphpad Software Inc., La Jolla, CA). For group mean waveforms, activity data for each rat were normalized relative to their daily means. The onsets of food anticipatory activity bouts were defined as the first 10 min bin within 4 h of mealtime in which activity exceeded 30 or 40 counts after an interval of 120 min during which this threshold was not exceeded. The lower threshold was used for rats with lower mean daily activity levels that exhibited premeal activity that failed to meet a 40 count criterion on many days. The same threshold was used for all conditions within each rat. Onsets were identified automatically using Circadia software. The duration of anticipation was defined as the difference in minutes between activity onset and mealtime. Proportionality between the duration of anticipation and the interval between lights-on and mealtime (a measure of the scalar property) was evaluated by calculating the ratio of anticipation duration to the interval. Differences in the timing of food anticipatory activity across days were evaluated statistically by repeated measures ANOVA, and post-hoc t-tests with Bonferroni corrections. Consistent with chronobiological conventions for nocturnal animals, time of day relative to the LD cycle is reported as ‘Zeitgeber Time’ (ZT), with lights-off designated as ZT12. In a 14∶10 LD cycle, lights-on is therefore designated ZT22. Group means are reported in the text and plotted with standard errors.

## Experiment 1. Two meals 10 h apart: meal omission and shift tests in LD and DD

### Introduction

We first quantified the duration and amount of wheel running prior to two daily meals beginning 3-h and 13-h after lights-on. We then conducted a series of meal omission tests in LD and DD, and a single 3-h meal shift test in LD. Predictions from single consulted clock, dual-FEO and interval (scalar) timing models are as follows. If anticipation of one or both meals is mediated or modulated by measuring elapsed time from LD transitions or the prior meal, then two predictions obtain. First, given the disparate light-food and intermeal intervals associated with the two mealtimes, the two bouts of anticipatory activity should differ in average duration, standard deviation of onset across days, and peak level [32]. Specifically, anticipation duration should scale with intervals relative to lights-on or prior mealtime. Second, anticipation to one or both meals should fail or exhibit significantly altered timing when LD or meal cues are absent. If anticipation of two daily meals is based on a single food-entrainable, continuously consulted clock, and the clock has measurable cycle-to-cycle variability (analogous to the light-entrainable SCN pacemaker, which exhibits variability in phase or period across days) then over many days, the duration of the two bouts of anticipation should be positively correlated (i.e., onsets should move forward and backward in parallel, in response to stochastic or induced changes in clock phase). If anticipation is based on entrainment of two independent FEOs, then anticipation onsets are more likely to be uncorrelated. Also, a shift of one mealtime would be expected to shift one bout of anticipation without an immediate parallel movement of anticipation to the next meal.

### Procedures

**Figure 1:**
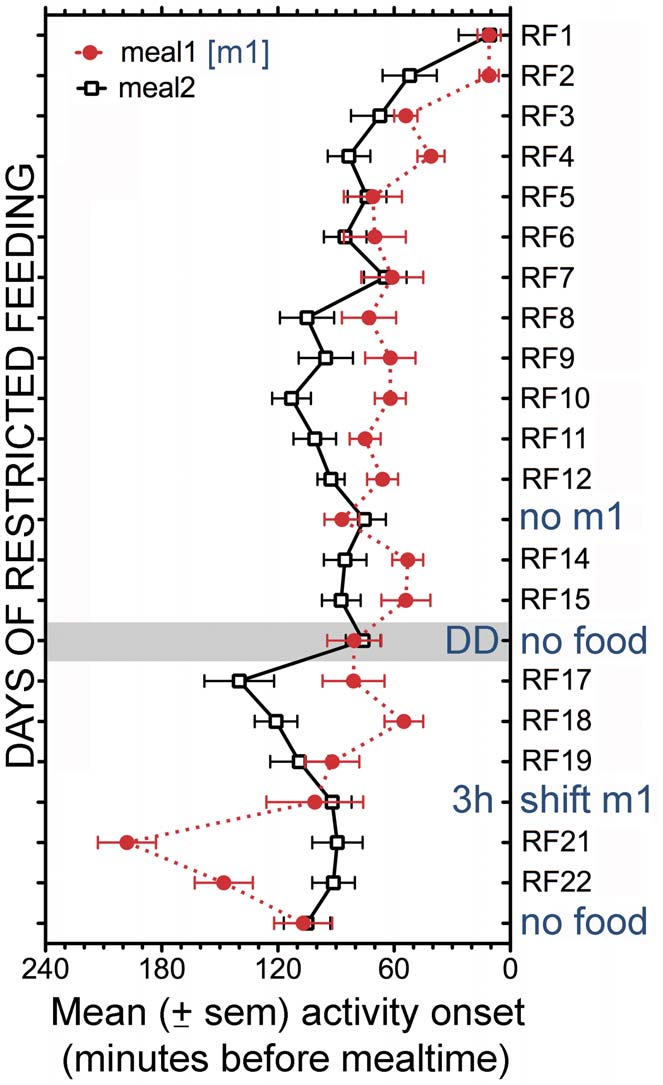
Group mean (± SEM, N = 15 rats) anticipatory wheel running onsets in minutes prior to meal 1 (m1; red closed circles, dotted curve) and meal 2 (open squares, black solid curve) during each day of restricted feeding (RF) in **Experiment 1** **.** Mealtime 1 and 2 began 3-h and 13-h after lights on (LD 14∶10). Meal 1 was omitted on day 13, both meals were omitted and the lights were left off on day 16, meal 1 was delivered 3-h early on day 20 and both meals were omitted on day 23.

Rats (N = 15) were acclimated to the 14∶10 LD cycle for 6 weeks and to the running wheels for the last 3 of these weeks. The rats were weighed and then food deprived for 37-h, beginning at lights-off. Food (12 g of 5001 rodent chow pellets) was then provided twice daily, 3-h after lights-on (ZT1, designated Mealtime 1 or the morning meal) and 1-h before lights-off (ZT11, designated Mealtime 2 or the afternoon meal). Any remaining pellets were removed after 1-h. On day 13 of restricted feeding (RF13) meal 1 was omitted. On day RF16, both meals were omitted and the lights were not turned on. On day RF20, meal 1 was provided 3-h early for that day only. On day RF23, both meals were omitted a second time, in LD. The sequence of conditions are illustrated in Figure 1.

### Results

#### Mean duration and variability of anticipation

**Figure 2:**
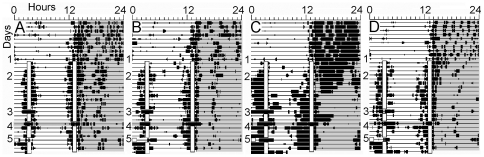
Wheel running activity of four representative rats in Experiment1. Each line represents 24 h, with time of day plotted left to right in 10 min bins. Time bins during which wheel counts were registered are denoted by heavy bars. Meals are indicated by opaque bars. Experimental conditions are numbered to the left of each panel: 1 = 37-h food deprivation, 2 = restricted feeding days, 3 = meal 1 omitted, 4 = both meals omitted in constant dark, 5 = meal 1 provided 3-h early.

**Figure 3:**
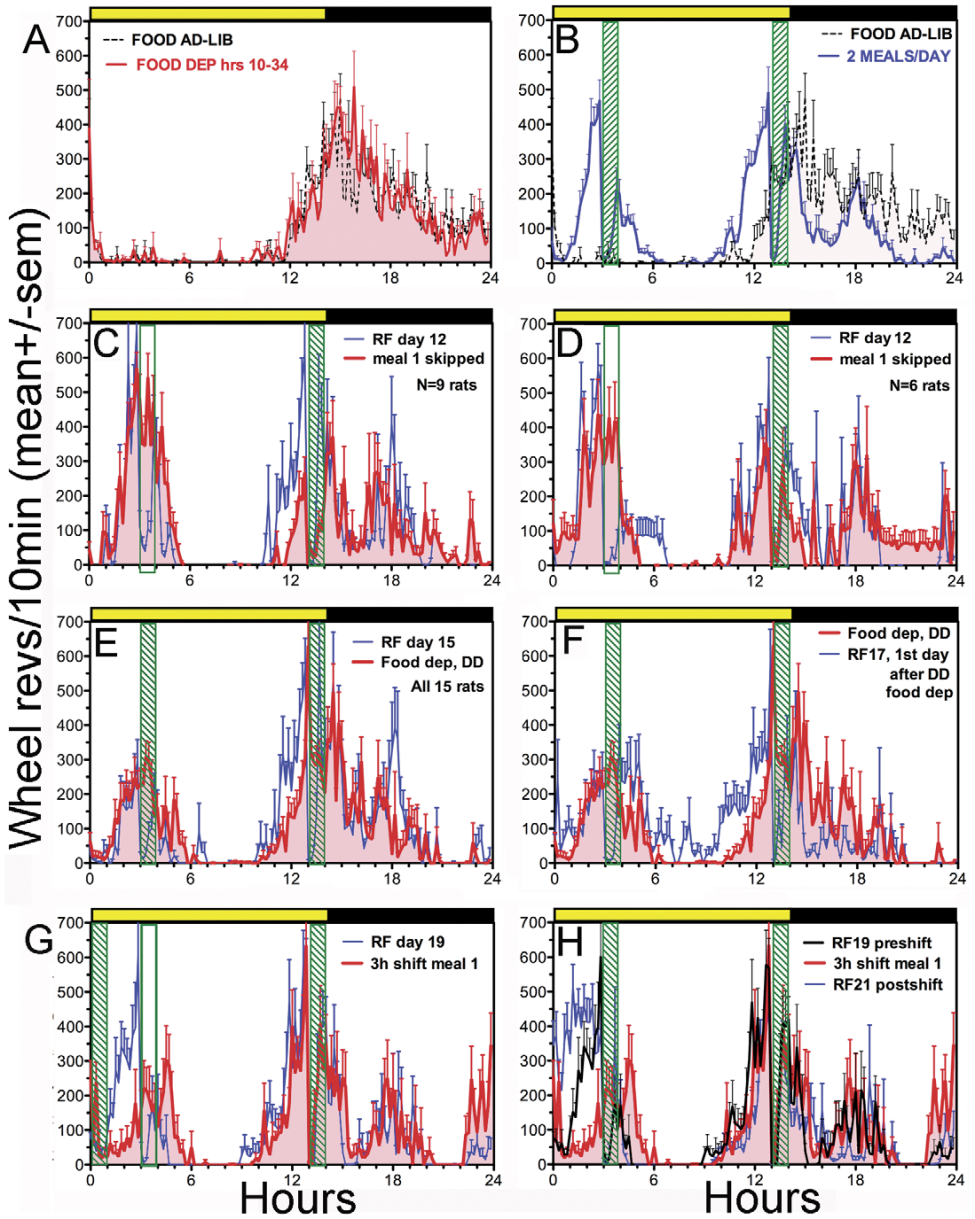
Group mean (± sem) waveforms of normalized wheel running activity in **Experiment 1** **.** Time is plotted in 10 min bins from lights-on (Hour 0, yellow bar). Meal times are denoted by green stripped vertical bars (hollow when meals were skipped). A. Last 4 days of ad-lib food access (dashed line) and 37-h food deprivation (red solid line, shaded) prior to initiation of 2-meal restricted feeding (RF) schedule. B. Ad-lib (dashed line) and RF days 8–12 (blue, solid line). C. RF day 12 (blue line) and Meal 1 omission day (red line, shaded), n = 9 rats showing delayed onset of anticipation to Meal 2. D. RF day 12 (blue line) and Meal 1 omission day (red line, shaded), n = 6 rats showing no change in anticipation onset. E. RF day 15 (blue line) and total food deprivation day with lights-off (DD, red line, shaded). F. Total food deprivation day in DD (red line, shaded) and first day of RF after food deprivation (blue line). G. RF day 19 (blue line) and 3-h shift of Meal 1 (red line, shaded). H. RF day 19 (black line), 3-h shift of Meal 1 (red line, shaded), and day after 3-h shift of Meal 1 (RF21, blue line).

Group mean onsets for each bout of food anticipatory activity on each day of the experiment are illustrated in Figure 1. There was a main effect of day on the onset of activity prior to Mealtime 1 (F_(27,378)_ = 8.68, p<.0001) and Mealtime 2 (F_(26, 364)_ = 10.1, p<.0001). Prior to food restriction, wheel running activity was predominantly nocturnal (Fig. 2A, B, C, D; Fig. 3A). When food was removed for 37-h prior to scheduled daytime feeding, the onset and level of nocturnal activity were remarkably stable, indicating that the LD-entrained circadian pacemaker was not acutely shifted by food deprivation (Fig. 3A). When food was provided for 1-h twice daily, at ZT1 and ZT11, running was evident in all rats prior to both meals by day RF3. Group mean activity waveforms (Figs. 3B; S1), generated by averaging across days RF8-12 prior to the first meal omission test, reveal two prominent bouts of food anticipatory running that differed significantly in mean duration (68±8 min Vs 101±9 min, for Mealtimes 1 and 2, respectively; t_(14)_ = 6.51, p<.0001). To determine if the duration of anticipation to the two mealtimes scaled with the intervals between lights-on and mealtime (180 min and 780 min for Mealtimes 1 and 2, respectively), the ratio of the mean anticipation bout length to the appropriate interval was calculated for both meals, for each rat. This ratio is a modification of the so-called coefficient of variance (CV) and should be equivalent for each mealtime if the rats measure intervals from lights-on to mealtime. The two ratios were instead markedly different (group mean ratios of .56±.05 for meal 1 anticipation, and .09±.02 for meal 2; t_14)_ = 10.93, p<.0001). The ratios were similarly discordant when the intermeal interval from Mealtime 1 to Mealtime 2 was used as the numerator for the second bout of anticipation, to test the possibility that rats timed meal 1 relative to lights-on, and meal 2 relative to meal 1 (600 min; CV = .11±.02).

The scalar property of interval timing also predicts a decreased peak level and increased variability of anticipatory activity onsets to Mealtime 2 relative to Mealtime 1 (commensurate with the longer interval from lights-on or from Mealtime 1). Contrary to these predictions, the two bouts of anticipatory activity rose to the same terminal peak (Figs. 3B, S1) and exhibited equivalent within-subject variability of onset times across days (standard deviations of activity onsets averaged 27±3 min prior to Mealtime 1 and 25±3 min prior to Mealtime 2; t_(14)_ = 0.56, p = .58).

A plot of premeal activity for each rat on a single day (RF12) suggests that the shape of the waveforms obtained by averaging across animals and across days (RF8-12) is representative of individual rats on single days (Fig. S1A). Regression lines fit to activity counts for each minute of the last hour before mealtime exhibited a positive slope in 13 of 15 rats at both mealtimes (Fig. S1B,C insets). Thus, the rate of anticipatory wheel running on individual days exhibits a gradual acceleration prior to feeding, rather than a step change from a low to a high rate of running, as is sometimes seen with short-interval timing [32].

To minimize disturbances, the rats were weighed only twice during the study. On the last day of ad-lib food access, the rats weighed on average 539±9 gms. After 9 days of restricted feeding, body weights were reduced on average by 4±1%. Body weights on this day were negatively correlated with the duration of food anticipation on that day. The association was stronger for Mealtime 1 anticipation (r = −.58, p = .02) than for Mealtime 2 anticipation (r = −.30, p>.05).

#### Meal omission tests

When meal 1 was omitted on day RF13, all 15 rats exhibited anticipation of the second meal. The onset of anticipatory activity to Mealtime 2 was modestly delayed relative to the previous 5 day average (26±8 min late; paired t_(14)_ = 3.13, p<.05). Closer inspection revealed two subgroups of rats, a group of 9 in which anticipation was late (Fig. 3C) and a group of 6 in which anticipation was virtually unaltered (i.e., within one 10 min bin; Fig. 3D). Although delayed, anticipation in the group of 9 was still significant relative to their baseline activity onsets (44±9 min, t_(14)_ = 4.86, p<.005). On day RF16, after two days of receiving both meals, the lights were left off and both meals were omitted. Relative to day RF15, anticipation of Mealtime 1 began 27±21 min early while anticipation of Mealtime 2 began 13±8 min late, and neither difference approached statistical significance (Fig. 3E).

On day RF17 the LD and feeding schedules were reinstated. Despite >1.5 days (37-h) without food, neither the onset nor the peak level of anticipation to Mealtime 1 was altered relative to the deprivation day or to day RF15 (Figs. 1, 3E, F). By contrast, the onset of Mealtime 2 anticipation was advanced in 11 of 15 cases (Figs. 1, 3E–F; group mean change = 64±15 min, t_(14)_ = 4.32, p<.003). Over the next 2 days, Mealtime 2 anticipation shifted back toward its original onset time (Fig. 1, days RF18-19).

#### Meal shift test

On day RF20, the morning meal was delivered 3-h early, at light onset. Despite the early feeding, most of the rats exhibited some activity at the clock times normally associated with the beginning and ending of meal 1 (Fig. 3G). The timing of anticipation to Mealtime 2 on this day was not significantly altered relative to the preceding day (RF19, mean change = 17±14 min, t_(14)_ = 1.20, p = 0.25). On the following day (RF21) the original morning mealtime was reinstated. The morning bout of anticipation on that day was markedly advanced (107±15 min, t_(14)_ = 7.16, p<.0001; Figs. 1, 3H), whereas there was again no change in the timing of anticipation to Mealtime 2 (mean change = −3±11 min). Over the next 2 days (Fig. 1, RF22-23), Mealtime 1 anticipation shifted back towards its original onset time, while Mealtime 2 anticipation remained stable.

#### Correlation of anticipation onsets across all days

To further assess the relationship between the two bouts of food anticipation during restricted feeding, Pearson correlation coefficients were calculated for each rat. Daily variations in onset times of the two activity bouts were not significantly related in any rat, across all 23 days (average Pearson r = .19±.06) or from days 8–23 (omitting the first week, when both bouts of anticipation were emerging in parallel; average Pearson r = .00±.07). The correlation coefficient approached zero for the group mean data (r = .01; Fig. 1).

### Discussion

The results of Experiment 1 provide no evidence that interval timing contributes to the timing of food anticipatory activity in rats fed two daily meals in the light period. Although the longer duration of Mealtime 2 anticipation relative to Mealtime 1 anticipation is in the direction predicted by scalar timing, the ratio of the duration of anticipation to the intervals between lights-on and mealtimes did not exhibit proportionality, a hallmark property of interval timing. Also, the within-subject variability of anticipation bout duration across days did not differ between Mealtimes 1 and 2 (variability across days should also scale with interval duration) [32]. Crucially, anticipatory activity to both meals persisted with little or no shifting when one or both meals were omitted, in either LD or DD, leaving no external cues available. Finally, shifting the morning mealtime failed to alter the timing of anticipation to the afternoon meal, ruling out endogenous feeding-related cues as stimuli that might actuate an interval timer for measuring elapsed time between meals. Taken together, the results demonstrate that the onset of food anticipatory wheel running in this 2-meal paradigm is under the control of one or more circadian clocks, and does not involve measurement or memory of elapsed time between external events.

Other results of Experiment 1 tend to favor a dual-FEO model of 2-meal anticipation over a single continuously-consulted-clock model. On two occasions, anticipation of one meal was acutely shifted without a change in the timing of the next bout of anticipation. A rapid shift of meal timing after a single event (e.g, one early meal, or one refeeding after a 37-h food deprivation) is predictable if each bout of anticipation is timed by a separate oscillator that is entrained (and thus can be acutely shifted) by one meal, analogous to resetting of the SCN pacemaker by a single light or dark pulse. If anticipation of both meals was based on a single food-entrained oscillator (functioning as a continuously-consulted-clock), then both bouts of anticipation would be expected to shift in lock step on the first cycle after one bout has shifted. Across all 23 days of the feeding schedule, the daily onsets of the two bouts of anticipation were not significantly correlated within subjects or in group data, a property more consistent with two independently entrained FEOs rather than a single consulted clock.

## Experiment 2. Two daytime meals 10-h apart: assessment of SCN phase

### Introduction

If rats use separate circadian oscillators to anticipate 2 daily meals, could one of these be the LD-entrained SCN? Our strategy of scheduling the second daily meal 1-h prior to lights-off, to maximize the intermeal interval, creates the impression that nocturnal activity may have phase shifted, raising the possibility that activity prior to Mealtime 2 represents the onset of nocturnal activity driven by the SCN. This interpretation would require an ∼3-h phase advance shift of the SCN, despite continued exposure to a 24-h LD cycle. Several well established facts argue against this interpretation. First, it has been shown repeatedly that in LD-entrained rats, the SCN is not phase shifted by daytime feeding schedules [33]–[37]. Food-restricted mice may show a phase shift of nocturnal activity onset, but only on caloric restriction schedules that markedly reduce body weight (∼20%, compared to ∼4% in the present study) [38]. In that study [38], no shift of *per1* or *Bmal1* clock gene rhythms in the SCN was observed, suggesting that clock phase was not altered and that severe caloric restriction affects daytime activity downstream from the circadian clock. Second, during daytime restricted feeding, SCN-driven nocturnal activity is typically decreased in rats [4], and SCN output during the light period is actively inhibited [39]. Third, abrogation of SCN function in rats by lesion [40] and in mice by clock gene mutation [41] enhances the magnitude of food anticipatory activity. Finally, SCN-ablated rats robustly anticipate and can discriminate 2 daily mealtimes [15], [16]. Thus, the rat SCN in LD is not shifted by daytime feeding, its output is if anything inhibited during restricted feeding, and a mechanism for anticipating 2 daily meals is present elsewhere in the brain. These facts do not support a role for the SCN as a clock driving daytime food anticipatory rhythms. Rather, the prevailing interpretation is that FEOs and the light-entrainable SCN are distinct timing mechanisms that compete for control of activity, with FEOs dominating during scheduled feeding, and the SCN dominating when there are no constraints on feeding.

To test this argument, we conducted an additional experiment to assess the phase of the SCN following daytime restricted feeding. Rats in LD 14∶10 were subjected to the same 2-meal feeding schedule, or were fed ad-lib, and then both groups were provided food ad-lib in DD. Food anticipatory activity rhythms persist during total food deprivation, but are markedly attenuated or absent on the first day of ad-lib food access, which combined with DD should reveal the true phase of SCN-driven nocturnal activity.

### Procedures

Sixteen male Sprague Dawley rats naïve to restricted feeding schedules were randomly assigned to a food restriction group (N = 8) or an ad-lib fed group (N = 8). The rats were first acclimated to the running wheel cages for 2 weeks with ad-lib food access. The food restriction group was food deprived for 37-h, and then fed twice daily for 1-h, beginning at ZT1 and ZT11, for the next 15 days. After the last meal at ZT11, food was provided ad-libitum in constant dim red light (DDr, <1 lux) for 10 days.

### Results and Discussion

By contrast with the results of Experiment 1, food anticipatory wheel running was less robust in most rats in Experiment 2. Nonetheless, all 8 food restricted rats exhibited some anticipation of Mealtime 1, and 5 of 8 also showed significant anticipation of Mealtime 2 (e.g., Fig. S2A), with an average bout duration similar to that evident in Experiment 1 (108±8 min). Activity onsets on each of the first two days of DDr were compared with onsets averaged over the week prior to restricted feeding, and these differences were then compared between groups (Fig. S2C). The 5 rats that anticipated Mealtime 2 were treated as a separate group for contrast with the ad-lib fed rats. The onset of activity on the first day of DDr did not differ from nocturnal activity onsets during the last week of ad-lib food access in this subgroup of 5 rats (*t_(4)_* = 1.23, p = .30) or in the ad-lib fed group (*t_(7)_* = 0.65, p = .53). The onset of activity on the first day of DDr, expressed as differences from the onsets prior to restricted feeding, also did not differ significantly between the ad-lib fed rats and the food restricted rats (all 8, t*_(14)_* = 0.41, p = .68; subgroup of 5, t*_(11)_* = 0.09, p = .92). These results provide no evidence that the SCN was phase advanced by the daytime feeding schedules.

## Experiment 3. Two daytime meals, 7-h apart

### Introduction

The 10-h interval between mealtimes 1 and 2 in Experiments 1 and 2 was chosen to minimize ambiguity in identifying distinct bouts of activity associated with each mealtime during meal omission tests, which is the most important criterion for determining whether the two bouts are under independent circadian control. A wide interval should also facilitate detection of differently phased circadian clock gene rhythms in different brain regions or peripheral organs, if separate bouts of anticipation are mediated by different groups of circadian clock cells, functioning as independently entrained FEOs. In earlier work, total food deprivation tests conducted when two daily meals were 5-h apart revealed only a single bout of activity extending across the two mealtimes [7]. In Experiment 3, we examined an interval of 7-h, toward defining discrimination limits for 2-meal timing. We also explored the use of 1 and 2 day food deprivation tests and a food-bin activity measure as methods for uncovering meal timing in rats that exhibit weak or no food anticipatory wheel running, as was observed in Experiment 2.

### Procedures

Two cohorts of rats entrained to LD 14∶10 were housed in wheel running cages as in Experiment 2, with food provided in an external food bin accessed via a window. After two weeks of ad-lib food access, the rats were food deprived for 37-h and then received food (powdered chow in corn oil) twice daily, 3-h (ZT1) and 10-h (ZT8) after lights-on. Powdered chow was used so that rats could not remove it from the food bin, and would therefore focus activity on the food bin. The first cohort of rats (N = 8) were food restricted for 41 days, during which the morning meal was omitted once (RF day 22), both meals were omitted for one day in LD (RF25) and for two days in DD (RF29-30), and the morning meal was advanced by 3-h once (RF34). The second cohort (N = 16) were food restricted for 34 days, during which the afternoon meal was omitted once (RF7), the morning meal once (RF17), both meals for 2 days in DD (RF21-22) and both meals for 1 day in LD (RF31).

### Results and Discussion

**Figure 4:**
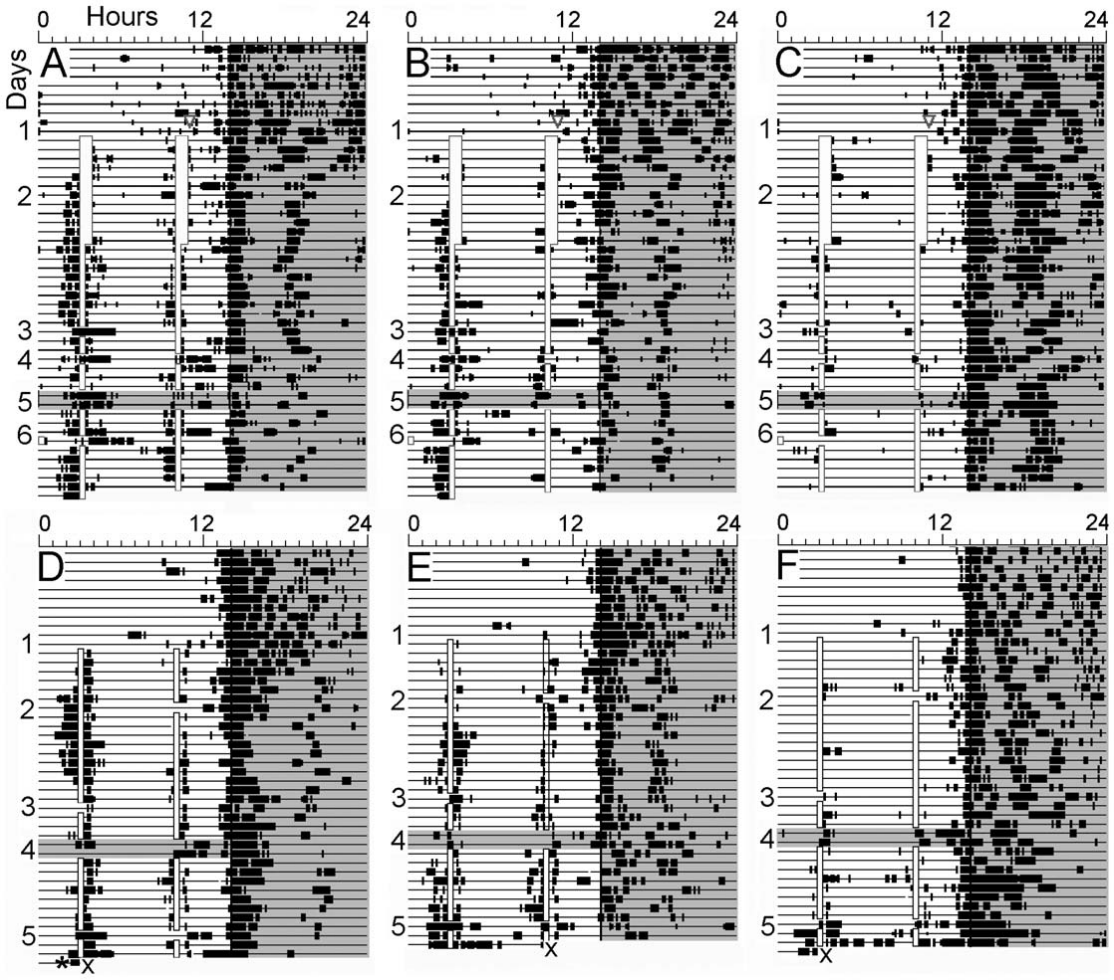
Wheel running of representative rats in **Experiment 3** **. Panels A–C. Rats from cohort A.** Experimental conditions are numbered to the left of each actogram: (1) 37-h food deprivation. (2) Two daily meals, denoted by opaque vertical bars. (3) Meal 1 omitted. (4) Both meals omitted. (5) Both meals omitted for 2 days in DD. (6) Meal 1 advanced by 3-h. Panels D–F. Rats from cohort B. Experimental conditions: (1) 37-h food deprivation. (2) Meal 2 omitted. (3) Meal 1 omitted. (4) Both meals omitted for 2 days in DD. (5) Both meals omitted in LD.

**Figure 5:**
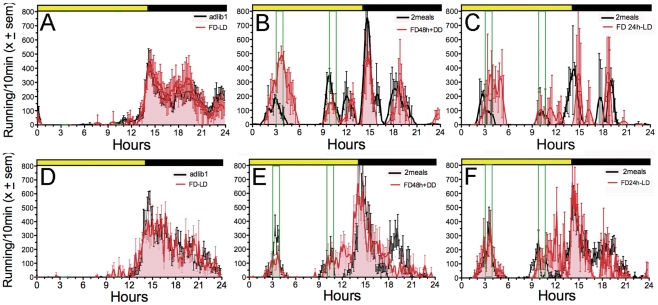
Group mean waveforms of wheel running activity in rats from Cohort A (panels A–C) and Cohort B (panels D–F). Panels A,D: ad-lib food access (grey shading) and 37-h food deprivation prior to restricted feeding (red curve). Panels B,E: Restricted feeding (RF) days (black line) prior to 2 days food deprivation (FD, red line) in constant dark (DD). Panels C,F: RF days (black line) prior to omission of both meals for one day (red line) in LD. Other plotting conventions as in Figure 1.

Food anticipatory wheel running was again less robust in this experiment by contrast with Experiment 1. Of the 24 rats, 12 showed anticipatory activity to Mealtime 1 on most days, with variable expression of anticipatory activity to Mealtime 2 (e.g., Fig. 4A,B,D,E). The other 12 rats showed little anticipation of either meal on most days (e.g., Fig. 4C,F). When anticipation was present, in most cases it persisted during meal omission tests in LD and DD, with no systematic shift in the time of onset (Fig. 5). A notable feature of the results was the appearance of activity before or during the scheduled mealtime during meal omission tests in rats that did not exhibit food anticipatory activity on the immediately preceding days (e.g., Fig. 4C,F). This implies that meal timing may be operational even when meal anticipation is not expressed on most days, and that the timing process may be more precise than is suggested by the typical duration of anticipatory activity.

**Figure 6:**
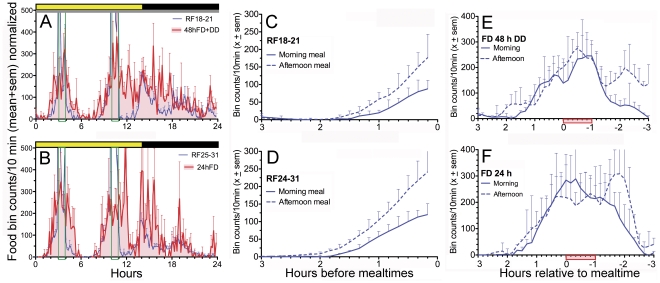
Anticipatory food-bin activity in **Experiment 3** **(cohort B, N = 16).** A. Group mean activity waveform during restricted feeding (RF) days 18–21 (blue curve) immediately prior to 2 days of total food deprivation (FD) in constant dark (DD) (red curve and shading). B. Group mean activity waveform during RF days 25–31 (blue curve) immediately prior to 1 day of FD (red curve and shading). C. Food bin counts during each 10-min bin starting 3-h prior to the morning meal (Mealtime 1, solid blue curve) and the afternoon meal (Mealtime 2, dashed blue curve), from Panel A. Data normalized and smoothed. [D] Food bin counts prior to Mealtime 1 and 2 (from Panel B). E. Food bin counts from 3-h before to 3-h after Mealtime 1 (solid curve) and Mealtime 2 (dashed curve) during 2 days of FD. F. Food bin counts from 3-h before to 3-h after Mealtime 1 (solid curve) and Mealtime 2 (dashed curve) during 1 day of FD.

Food bin activity in the second cohort of rats (N = 16) was measured by a photobeam across the food-bin window, to determine if this might reveal anticipatory behavior in the absence of wheel running. This proved not to be the case, and overall the results conformed closely to the wheel data. Of 6 rats that showed little anticipatory running, pre-meal food bin activity was nearly absent in 4 cases and sporadic in the other 2, but, like running, was evident at mealtime during meal omission tests. The other 10 rats showed anticipatory food bin activity to both meals on most days, and the timing of these bouts was not significantly changed during meal omission tests in LD (meal 1, t_(9)_ = 1.2, p>.1; meal 2, t_(9)_ = 1.13, p>.1) and DD (meal 1, t_(9)_ = 1.9, p>.1; meal 2, t_(9)_ = 0.38, p>.1) (Fig. 6). As was observed for wheel running in Experiment 1, the duration of anticipatory food bin activity was greater prior to meal 2 than meal 1 (e.g., for restricted feeding days 24–31, 60±7 min vs 79±6 min, t_(9)_ = 2.66, p<.02, Fig. 6C,D). During meal omission tests, activity peaked during the expected mealtime, and was approximately symmetrical around this peak (Fig. 6E,F). The width of the distributions of activity associated with each mealtime were quite similar, and clearly not proportional to the different intervals between lights-on and mealtime or between mealtimes, as would be predicted by interval timing models.

The average amount of anticipatory food bin activity to Mealtime 1 and 2 increased by factors of 2.5 and 1.8, respectively, during the week following the first 48 h meal omission test. This suggests a possible effect of weight loss incurred during fasting. Body weights were found to discriminate the group of anticipators from non-anticipators (F_(1,28)_ = 8.68, p = 0.0064); compared to the 10 rats that anticipated daily meals, the 6 non-anticipators weighed more both at the beginning (548±8 g vs 515±10.4, p<.05) and the end (559±9 vs 517.3±14, p<.05) of restricted feeding.

### General Discussion

These experiments were designed to probe formal properties of food anticipatory rhythms in rats maintained on two daily meals in the light period, and to assess quantitatively whether anticipation of two daily meals involves both circadian and interval timing processes [21]. The results in aggregate provide little support for this proposition, and instead indicate that when food is available at two fixed times of day, anticipatory behavior is under circadian clock control. The persistence (or appearance) of food anticipatory activity at scheduled mealtimes during meal omission tests (Experiments 1 and 3) is consistent with predictions of 2-oscillator and consulted clock models, but not interval timing models. Shifting of one bout of food anticipation without parallel shifting of anticipatory running to the next meal is consistent with predictions of a 2-oscillator model, as is the lack of association between the timing of the two bouts of anticipation across all 23 days of restricted feeding in Experiment 1. These results are readily interpretable within the framework of a dual-FEO model, and constitute a novel set of observations that complement existing but limited evidence for dual FEOs derived from experiments in which two meals were provided with slightly different periodicities [6], [8].

The failure of the two daily bouts of anticipatory activity to meet criteria for interval timing, in particular the lack of proportionality between the bout durations and the intervals between lights-on and mealtimes or between the 2 mealtimes, as well as the similar degree of variability of the two bout durations within-animals across days, differs from the results of a single meal study, in which proportionality of anticipation duration to single daily meals 3-h or 7-h after lights-off was observed [26]. It is possible (although not likely) that lights-off is a more salient cue than is lights-on to support interval timing in the multi-hour range. Alternatively, rats may be more inclined to measure intervals when acquisition of food is contingent on operant behavior, such as lever pressing or nose pokes, as in [26] and in most other studies of short-interval timing in rodents. General locomotor activity measures, such as wheel running or exploratory activity, may primarily reflect circadian processes such as oscillator entrainment or clock consultation, whereas learned operant behaviors may be more likely to come under joint control of circadian and interval timing processes. Support for this interpretation is provided by an earlier study, which showed interval timing effects in anticipatory lever pressing but not in general locomotor activity measured concurrently in rats maintained on a daily feeding schedule (Fig. 4 in [25]). Taken together, these results indicate that under some circumstances, food anticipatory activity can be jointly regulated by circadian and interval timing, but that this is dependent on the food delivery procedures (free-food Vs. working for food) and behavioral measures (operant Vs. non-operant). To our knowledge, no study of intact rats or mice has been able to demonstrate anticipation of one or two daily meals based exclusively on external, cue-dependent interval timing.

In Experiment 1, all of the rats exhibited robust anticipation of both meals, but in the follow-up experiments, anticipation was notably weaker and more variable in the number of rats anticipating one or both meals, and in the amount of premeal running in those that did anticipate. The cause of this variability across experiments is unclear, but could involve procedural or physiological variables. Although the running wheel cages were the same in all of the experiments, in Experiment 1 the cages were housed in individual isolation boxes, whereas in the other experiments the cages were in open cabinets. Also, in Experiment 3 food was provided in an external food bin accessed through a small window. Whether these differences played any role in the amount of anticipatory running is uncertain. One previous study found an effect of housing, albeit in a different species and in the opposite direction; mice in isolation boxes failed to anticipate a daily meal, whereas the same mouse strain did anticipate meals when housed in open racks [42]. A physiological variable that can affect the expression of food anticipatory activity is body weight, as rats made obese by a cafeteria diet exhibit less anticipation [43], while obese leptin receptor-deficient Zucker fatty rats and obese leptin-deficient *ob/ob* mice exhibit greater anticipation [44], [45]. Body weight was associated with anticipatory behavior in both Experiment 1 and 3, in the expected direction (greater weight associated with less anticipation), but the starting body weights of the rats did not differ across studies. Despite the absence of anticipation in a substantial number of rats in Experiments 2 and 3, the meal omission tests in DD revealed evidence of meal timing behavior in most cases. This suggests that factors associated with body weight modulate the expression of food anticipatory activity downstream from the anticipatory timing mechanism.

In addition to metabolic factors, the expression of food anticipatory activity is likely also affected by ambient lighting. Some rats in Experiment 3 that failed to exhibit food anticipation to meal 1 in LD, did exhibit anticipation of meal 1 on the first day of DD, prior to meal omission. Light is known to suppress locomotor activity in nocturnal rodents [46], and evidently this effect is not entirely superseded by daytime restricted feeding schedules. Light and metabolic factors may modulate thresholds gating expression of clock-controlled anticipatory behavior.

The neural mechanisms by which animals coordinate foraging activity with daily feeding opportunities remain to be elucidated. While phenomenological evidence supports the concept of a dual FEO system underlying 2-meal timing in rats, the validity of this model will require confirmation at the cellular level, by the observation of independently phased circadian oscillations in one or more neuronal groups that could, in principle, be manipulated to evaluate a role as independent driving oscillators for separate bouts of anticipatory activity. This task is complicated by continued uncertainty about the location of circadian oscillators critical for food anticipatory rhythms, and about the role of known circadian clock genes in the generation of these rhythms [19], [20], [22], [47]–[50].

## Supporting Information

Figure S1 **Group mean (± SEM) anticipatory wheel running in** **Experiment 1** **.** **A.** Overlay of running activity in each 10 min bin beginning 4-h prior to Mealtime 1 (solid line) and Mealtime 2 (dashed line), on restricted feeding (RF) days 8–12. B–C. Running activity during each 10 min bin beginning 3-h before Mealtime 1 (B) and Mealtime 2 (C), for each rat on day RF12. The inset panels illustrate the results of linear regression line fits to the last hour of activity in 1 min time bins, for each rat.
(TIF)

Figure S2 **Wheel running activity of representative rats in** **Experiment 2** **.** A. Rat subjected to the 2-meal daytime restricted feeding schedule. Mealtimes denoted by vertical open bars. B. Rat fed adlib. Light-off denoted by shading. Other plotting conventions as in Figure 2. C. Group mean activity onsets (± SEM) of food restricted rats (upper 3 data points) and ad-lib fed rats (lower 3 data points), in minutes relative to activity onsets during the last week of ad-lib food access in LD (2 m RF, AL base 2), and on the first two days of constant dark (DD1 and DD2).
(TIF)
