# Supplementary figures and images for: Circadian Clocks for All Meal-Times: Anticipation of 2 Daily Meals in Rats
Source: PLoS One. 2012 Feb 15;7(2):e31772. doi: 10.1371/journal.pone.0031772 (PMC3280322; doi:10.1371/journal.pone.0031772)

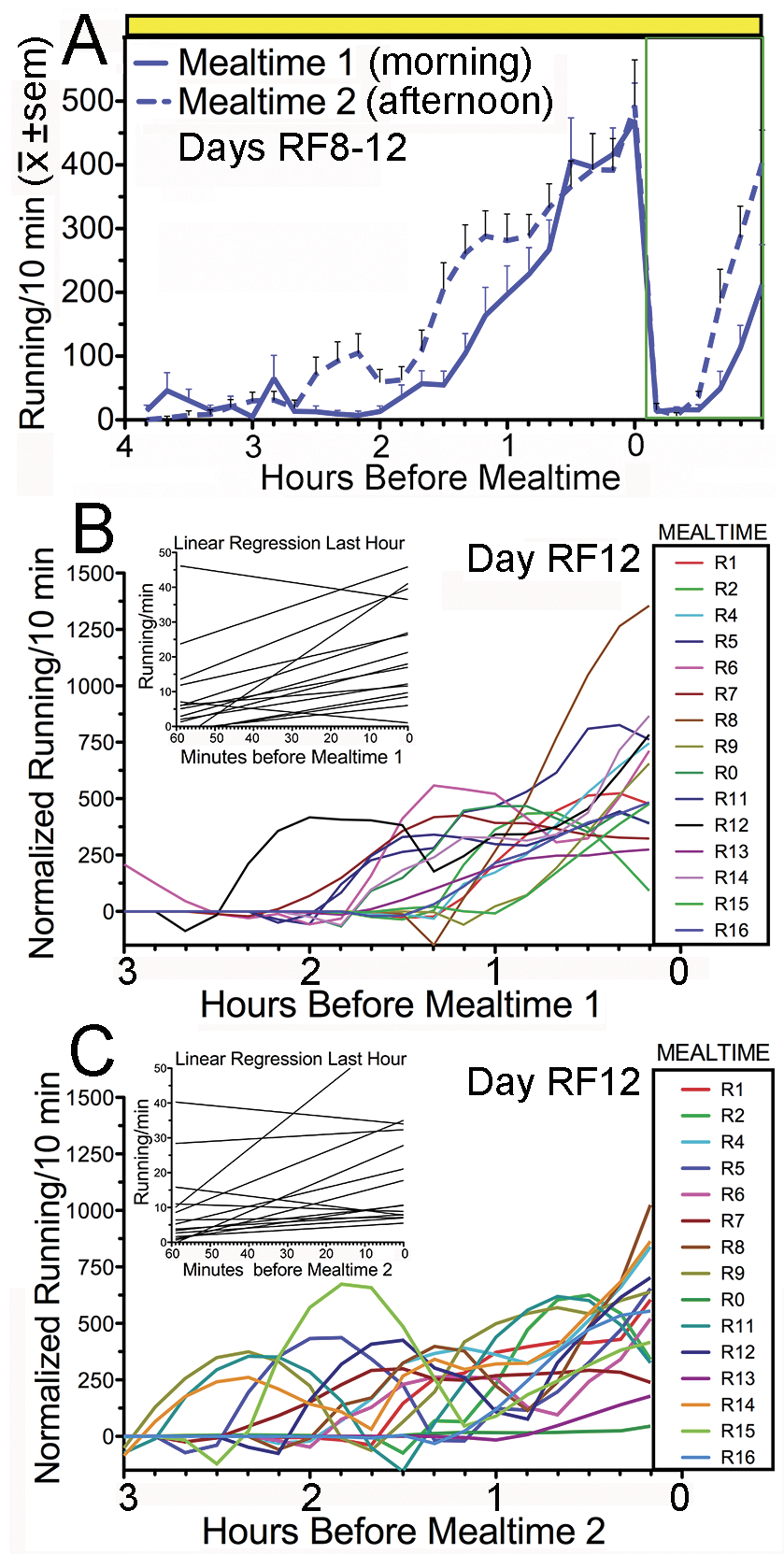

Supplement: Figure S1 — Group mean (± SEM) anticipatory wheel running in Experiment 1 . A. Overlay of running activity in each 10 min bin beginning 4-h prior to Mealtime 1 (solid line) and Mealtime 2 (dashed line), on restricted feeding (RF) days 8–12. B–C. Running activity during each 10 min bin beginning 3-h before Mealtime 1 (B) and Mealtime 2 (C), for each rat on day RF12. The inset panels illustrate the results of linear regression line fits to the last hour of activity in 1 min time bins, for each rat. (TIF) [file pone.0031772.s001.tif]

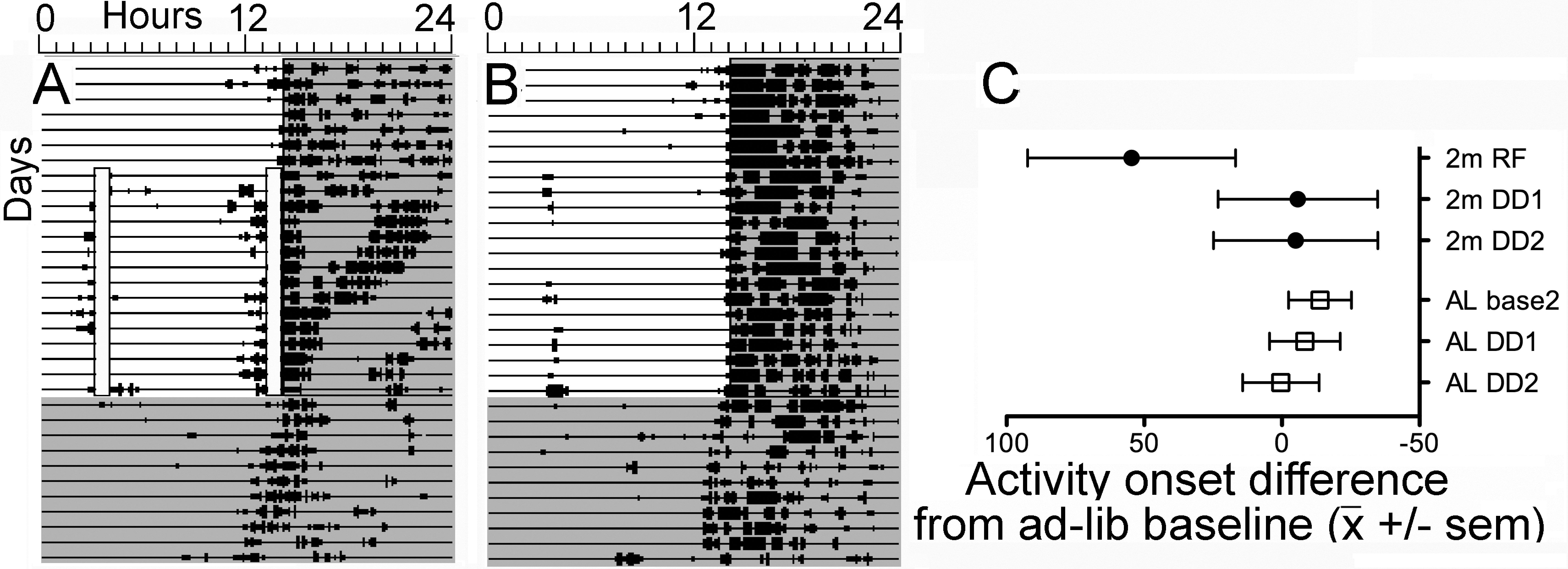

Supplement: Figure S2 — Wheel running activity of representative rats in Experiment 2 . A. Rat subjected to the 2-meal daytime restricted feeding schedule. Mealtimes denoted by vertical open bars. B. Rat fed adlib. Light-off denoted by shading. Other plotting conventions as in Figure 2. C. Group mean activity onsets (± SEM) of food restricted rats (upper 3 data points) and ad-lib fed rats (lower 3 data points), in minutes relative to activity onsets during the last week of ad-lib food access in LD (2 m RF, AL base 2), and on the first two days of constant dark (DD1 and DD2). (TIF) [file pone.0031772.s002.tif]
